# Dityrosine cross-linking and its potential roles in Alzheimer’s disease

**DOI:** 10.3389/fnins.2023.1132670

**Published:** 2023-03-22

**Authors:** Mahmoud B. Maina, Youssra K. Al-Hilaly, Louise C. Serpell

**Affiliations:** ^1^Sussex Neuroscience, School of Life Sciences, University of Sussex, Falmer, United Kingdom; ^2^Biomedical Science Research and Training Centre, College of Medical Sciences, Yobe State University, Damaturu, Nigeria; ^3^Department of Chemistry, College of Science, Mustansiriyah University, Baghdad, Iraq

**Keywords:** Alzheimer’s disease, amyloid-beta, tau, oxidative, dityrosine

## Abstract

Oxidative stress is a significant source of damage that accumulates during aging and contributes to Alzheimer’s disease (AD) pathogenesis. Oxidation of proteins can give rise to covalent links between adjacent tyrosines known as dityrosine (DiY) cross-linking, amongst other modifications, and this observation suggests that DiY could serve as a biomarker of accumulated oxidative stress over the lifespan. Many studies have focused on understanding the contribution of DiY to AD pathogenesis and have revealed that DiY crosslinks can be found in both Aβ and tau deposits – the two key proteins involved in the formation of amyloid plaques and tau tangles, respectively. However, there is no consensus yet in the field on the impact of DiY on Aβ and tau function, aggregation, and toxicity. Here we review the current understanding of the role of DiY on Aβ and tau gathered over the last 20 years since the first observation, and discuss the effect of this modification for Aβ and tau aggregation, and its potential as a biomarker for AD.

## Introduction

**FIGURE 1:**
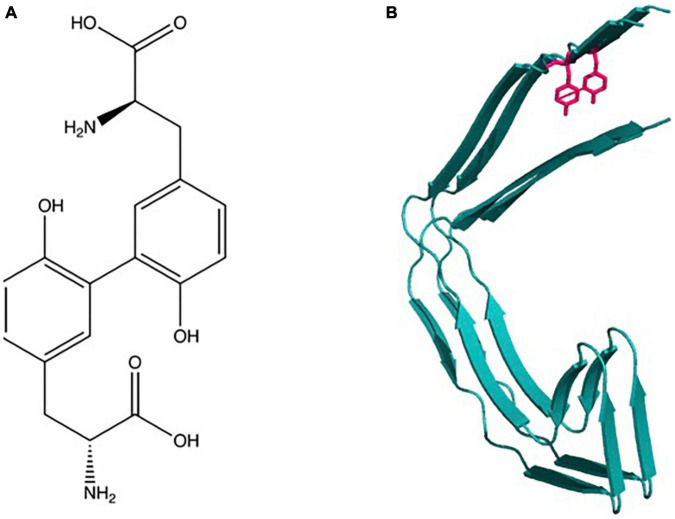
Dityrosine: **(A)** Chemical structure and molecular formula of dityrosine produced from two tyrosine amino acids. **(B)** Schematic shows depiction of crosslinking of two β-sheet rich protein molecules.

Dityrosine (DiY) is a covalent cross-link formed by ortho-ortho coupling between two tyrosine residues between carbons in the phenol ring (Figure 1). The general mechanism of its formation begins with the generation of a tyrosyl radical generated due to the removal of the hydrogen atom from the hydroxyl group on the phenoxy ring (Atwood et al., 2004; DiMarco and Giulivi, 2007). The two tyrosyl radicals then undergo radical isomerization followed by diradical coupling, and finally enolization resulting in DiY (Correia et al., 2012). The tyrosyl radical also has the capacity to react with another tyrosine to form tri-tyrosine and other high-order structures (Partlow et al., 2016).

Dityrosine was initially recognized by Gross and Sizer (1959) generated by peroxidase oxidation of tyrosine in the presence of H_2_O_2_. Previous work had established that peroxidase enzymes could catalyze the oxidation of phenols and aromatic amines by hydrogen peroxide (Elliott, 1932). Later, it was revealed that the diphenyl formation mechanism involved free radical generation as intermediates (Waters, 1952). Based on these observations, Gross and Sizer (1959) suggested that DiY formation was achieved through the generation of tyrosyl radical as an intermediate. Several studies revealed a native role of DiY in natural elastic materials and invertebrate tissues. DiY has been identified within resilin, the rubber-like protein found in arthropods (Andersen, 1964). This was the first documentation of the natural occurrence of this cross-linker in proteins and it was suggested to stabilize resilin through the formation of a stable three-dimensional network (Andersen, 1964). It was subsequently reported that DiY cross-links occur naturally in several elastic and structural proteins, including elastin, fibroin, keratin, cuticlin, and collagen (LaBella et al., 1967; Raven et al., 1971; Fujimoto, 1975; Waykole and Heidemann, 1976). In these proteins, DiY cross-links can contribute to increased mechanical strength and insolubility (Skaff et al., 2005).

Dityrosine has been shown to play a protective or stabilizing role in many proteins (Bailey, 1991; Kanwar and Balasubramanian, 1999). DiY has been found in fungal cell wall proteins (Smail et al., 1995) and in the fertilization envelope of the sea urchin egg where it may regulate the production of a hard fertilization membrane that blocks the entry of additional sperm (Foerder and Shapiro, 1977). In the large roundworm of the pig (*Ascaris suum*) it forms part of the structural components of the cuticle (Fetterer et al., 1993), and is involved in hardening of the mosquito egg chorion (Li et al., 1996). DiY has been suggested to contribute to spores’ resistance to lytic enzymes (Briza et al., 1990) and has been suggested to be involved in the biosynthesis of thyroxine and melanin (Bayse et al., 1972). For a detailed review of DiY in natural materials and mammalian tissues, the reader is referred to Partlow et al. (2016).

Oxidative environments can lead to the cross-linking of other amino acids as well as tyrosine and have been suggested to be associated with biological dysfunction. These include cross-links that form between Cys and Tyr, Trp, Lys, Ser, Phe; between Tyr and Gly, His, Trp and between His and His, Arg, Lys, and finally Trp-Trp. The most commonly reported cross-link in proteins is DiY (see Fuentes-Lemus et al., 2021 for extensive review of detailed chemical mechanisms). In this review, we focus on the literature relating to DiY formation in Alzheimer’s disease (AD) proteins Amyloid-β and Tau.

Alzheimer’s disease, Parkinson’s disease (PD), and other neurodegenerative diseases are classified as misfolding diseases reflecting the characteristic amyloid fibril pathology. Each disease is characterized by one or more proteins that form amyloid fibrils and in the case of Alzheimer’s disease, Aβ and Tau fibrils accumulate extracellularly and intracellularly, respectively. DiY cross-links in proteins have also been implicated in many diseases, including AD and PD (Souza et al., 2000; Atwood et al., 2004; Al-Hilaly et al., 2013, 2016, 2019), cystic fibrosis (Van Der Vliet et al., 2000), atherosclerosis (Leeuwenburgh et al., 1996), cataracts in the eye lens (Bodaness and Zigler, 1983; Wells-Knecht et al., 1993),, and acute myocardial infarction (Mayer et al., 2014). For example, DiY can be generated *in vitro* in samples of Aβ (Galeazzi et al., 1999; Atwood et al., 2004; Al-Hilaly et al., 2013; Maina et al., 2020b), α-synuclein (Souza et al., 2000; Al-Hilaly et al., 2016), and Tau (Reynolds et al., 2005; Maina et al., 2021, 2022b). However, there remains more to learn about the specific role played by this modification in these aggregation-prone proteins. Here, we review the studies that have explored the potential roles of DiY on Aβ, and Tau in the pathogenesis of AD.

## Amyloid beta

**FIGURE 2:**
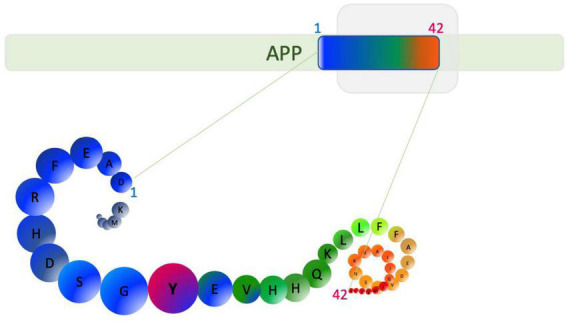
Aβ1–42 generation from amyloid precursor protein (APP). β-secretase cleavage beside aspartic acid and cleavage by γ-secretase can generate Aβ1–42 as well as other alternative length Aβ peptides. Amino acids are depicted as circles. Tyrosine (Y10) is able to form dityrosine cross-links and is highlighted in pink.

In his 1907 article describing the disease, Alzheimer’s post-mortem analysis of Auguste Deter, revealed intracellular neurofibrillary tangles and “minute miliary foci” deposited extracellularly as amyloid plaques (Alzheimer et al., 1995). This finding triggered a strong interest in understanding the biochemistry of these plaques and tangles, leading to the discovery that the plaques are predominantly made up of an amino acid peptide of about 40–42 residues and 4.2 kDa, now called Aβ (Glenner and Wong, 1984). The tangles were shown to be comprised of a hyperphosphorylated form of the microtubule-associated protein tau (Grundke-Iqbal et al., 1986a; Wood et al., 1986). Aβ peptide is synthesized from the processing of a single-pass integral membrane protein called amyloid precursor protein (APP) encoded by a gene located on chromosome 21, which has 18 exons, of which, exon 16 and 17 encode the Aβ peptide (Yoshikai et al., 1990). The processing of APP by β secretase 1 or β-site APP cleaving enzyme 1 (BACE 1) and γ-secretase (comprised of a complex including presenilin 1 and 2) leads to the generation of species of Aβ peptide (Figure 2).

The important role played by Aβ in AD pathogenesis led to the amyloid cascade hypothesis (Hardy and Higgins, 1992), based on the observation that early onset, familial forms of AD are associated with mutations in APP or presenilin 1 or 2 and that all appear to be associated with Aβ generation, deposition, or aggregation propensity (Levy et al., 1990; Van Broeckhoven et al., 1990; Chartier-Harlin et al., 1991; Goate et al., 1991; Hardy, 1991). The updated amyloid cascade hypothesis suggests that aggregation of Aβ from monomers to dimers, oligomers, fibrils, and eventually to plaques subsequently drives downstream changes, such as tau phosphorylation, cell loss, and dementia (Selkoe and Hardy, 2016). The amyloid cascade hypothesis has been supported by biomarker studies which show that changes in CSF levels of Aβ and its deposition into plaques appear decades before the onset of dementia (Jack et al., 2013).

Many pieces of evidence have subsequently shown that the oligomeric form of Aβ is the most toxic species, not fibrils, even though a consensus is lacking about the exact structure and composition of these soluble species (Benilova et al., 2012). Accumulated evidence shows that Aβ oligomers disrupt cellular function in cultured cells and animal models (Lambert et al., 1998; Lacor et al., 2007; Reddy and Beal, 2008; Wu et al., 2010; Li et al., 2011; Soura et al., 2012; Zhang et al., 2014; Fuchsberger et al., 2016; Marshall et al., 2016, 2020; Selkoe and Hardy, 2016; Biasetti et al., 2023). In the double-transgenic APP*^swe^*-Tau mouse, neuronal loss and activated astrocytes in the entorhinal cortex and the CA1 hippocampal subfield were found to correlate with the burden of Aβ oligomers (DaRocha-Souto et al., 2011). In human AD, soluble Aβ also correlates positively with the severity of dementia (McLean et al., 1999; Walsh and Selkoe, 2007). With the onset of the accumulation of Aβ oligomers, the novel AD mouse model—PS1V97L-Tg expressing the human PS gene with the V97L mutation, show synaptic alteration, tau hyperphosphorylation, and glial activation, hence supporting an early role for this Aβ species and their role in neurotoxicity (Zhang et al., 2014). Aβ oligomers alone have been shown to impair learning and memory in the pond snail *Lymnaea stagnalis* (Marshall et al., 2016; Ford et al., 2017). Collectively, these studies support the deleterious role of soluble Aβ, rather than fibrils (Zhu et al., 2011; Selkoe and Hardy, 2016). The role of the insoluble fibrils and plaques remains debated but it has been suggested that they may contain around them an equilibrium of both toxic oligomers and inert fibrils which may “spillover” to surrounding tissues to cause neuronal damage, and/or they may mediate toxicity by triggering neuroinflammation (Benilova et al., 2012).

## Dityrosine cross-linking in amyloid beta

Amyloid beta is known to become increasingly stable and insoluble as it self-assembles and is deposited in amyloid plaques in brain tissue that are highly protease resistant (Kheterpal et al., 2001). Aβ oligomers and fibrils formed *in vitro* and extracted from tissue show SDS-stability and resistance to proteolytic degradation (Wang et al., 1999; Walsh and Selkoe, 2007; Rambaran and Serpell, 2008; Shankar et al., 2008; Masters and Selkoe, 2012). However, such SDS resistance could arise from a number of different modifications, and/or experimental artifacts (Jomova et al., 2010; Sitkiewicz et al., 2014). The stability of the Aβ assemblies could arise from either the formation of a strong ionic complex or the formation of covalent cross-links, which may include transglutaminase (TGase)-induced cross-linking between the glutamyl side chains and the ε-amino groups of lysine; cross-linking generated by 4-hydroxynonenal (4-HNE) and DiY cross-linking (Siegel et al., 2007; Wilhelmus et al., 2009; Roberts et al., 2012; Al-Hilaly et al., 2013; Sitkiewicz et al., 2014).

Multiple studies suggest that DiY formation through Tyrosine (Y10) of Aβ could provide the observed stability of Aβ. DiY cross-linked Aβ has been found in the AD brain (de la Torre et al., 2018), around Aβ plaques and shown to occur *in vitro* (Al-Hilaly et al., 2013). We have also demonstrated that DiY cross-linked Aβ could be internalized into cells and found around amyloid plaques in the human AD brain, suggesting it may play a role in disease pathogenesis or as a marker of the disease process (Al-Hilaly et al., 2013). To understand the specific contribution of DiY cross-linking on Aβ and its contribution to AD, several studies have studied the influence of this cross-linking on Aβ assembly. *In vitro*, DiY formation has been primarily generated via metal-catalyzed oxidation (MCO), photooxidation, and enzyme-catalyzed mechanisms (Partlow et al., 2016). To induce DiY, copper has been mostly employed for MCO, ultra-violet light (UV) for photooxidation, and peroxidase for enzyme-catalyzed reactions (Yoburn et al., 2003; Atwood et al., 2004; Maina et al., 2020b). Early work from Galeazzi et al. (1999) showed that *in vitro* oxidation using H_2_O_2_/peroxidase induces DiY cross-linking on Aβ42 and suggested that this would promote Aβ aggregation in AD. This was the first evidence to suggest that DiY formation may play a role in stabilizing Aβ assemblies in AD.

Among the many processes that could lead to the formation of DiY, transition metal catalysis is particularly very relevant in the context of AD (Yoburn et al., 2003; Atwood et al., 2004). About 400 μM copper, 100 μM zinc, and 100 μM iron have been found around amyloid plaques, suggesting increased concentrations of these metals could be implicated in the pathogenesis of AD (Lovell et al., 1998; Suh et al., 2000; Bush, 2003). Many studies have demonstrated that copper Cu^2+^ ions coordinate Aβ via the three histidine residues; His6, His13, His14, and Tyr10 (Curtain et al., 2001; Atwood et al., 2004; Tickler et al., 2005). Coordination of Cu^2+^ ions to Aβ at these residues places Y10 in close proximity to the redox-active copper ion (Sarell et al., 2009; Viles, 2012; Gu et al., 2018). The Y10 has been shown to play a critical role in facilitating Aβ/Cu mediated H_2_O_2_ production (Barnham et al., 2004). Thus, it has been suggested that copper interactions with Aβ could be responsible for causing DiY cross-linking. This would be facilitated by the presence of H_2_O_2_ in the milieu which can be produced through Aβ’s ability to reduce Cu^2+^ (Yoburn et al., 2003; Atwood et al., 2004; Barnham et al., 2004). Indeed, Barnham et al. (2004) revealed that Aβ peptide coordinates Cu^2+^ to form an Aβ-Cu^2+^ complex, which in turn leads to the production of H_2_O_2_ catalytically in the presence of a reducing substrate such as ascorbate and through this process, tyrosine radicals are generated that are later coupled and result in Aβ aggregation. Atwood et al. (2004) showed that the incubation of Aβ with Cu^2+^ at a concentration lower than that in amyloid plaques led to the formation DiY cross-links on Aβ and the appearance of SDS-resistance Aβ oligomers, which is a characteristic feature of the neurotoxic Aβ extracted from the AD brain (Walsh and Selkoe, 2007). The authors also revealed that the addition of H_2_O_2_ to the reaction significantly promoted DiY formation compared to incubation with Cu^2+^ only, indicating that DiY formation becomes more favorable in an increased oxidative environment (Atwood et al., 2004). Smith et al. (2006) also demonstrated that the generation of the Aβ toxic species is modulated by both the Cu^2+^ concentration and the ability to form intermolecular histidine bridges.

There is an extensive literature on the impact of DiY cross-linking on Aβ assembly (Urbanc, 2021). Most of the early studies link DiY formation with Aβ aggregation (Galeazzi et al., 1999; Yoburn et al., 2003; Atwood et al., 2004; Zhang et al., 2017) or serves as a seed that promotes further aggregation (Ono et al., 2009). However, in the early *in vitro* studies in which DiY formation on Aβ was established, the aggregation of Aβ was not followed over time, especially using techniques like Thioflavine T(Th-T) fluorescence assay, to study the typical aggregation kinetics of Aβ. Collectively, recent literature suggests that DiY formation significantly stabilizes Aβ assemblies (Williams et al., 2016; Maina et al., 2020b), slows Aβ aggregation to fibrils (Al-Hilaly et al., 2013; Kok et al., 2013; O’Malley et al., 2014, 2016), or inhibits assembly into fibrils (Gu et al., 2018; Vázquez et al., 2019; Maina et al., 2020b). Some studies have linked DiY cross-linked Aβ with toxicity (Barnham et al., 2004; O’Malley et al., 2014; Williams et al., 2016) and others have shown that DiY inhibits self-assembly, and as a result, reduces or prevents Aβ toxicity (Maina et al., 2020b). To fully understand the exact roles of DiY on Aβ, it is thus important to consider the preparation method, buffer and temperature conditions, and Aβ sequence carefully since environmental conditions will have a significant effect on the eventual structures and the formation of any intermediate species (Table 1).

**TABLE 1:** Different oxidation conditions used in producing dityrosine in Aβ.

Aβ form and concentration	Method of DiY cross-linking	Results of oxidation	References
Aβ42 (25 μM)	Aβ in PBS pH 7 mixed with horseradish peroxidase (HRP) (30 μM)/0.3% H_2_O_2_ at 37^°^C for 6 h	Formation of Aβ42 dimers. DiY cross-linked Aβ42 remained soluble. Aggregation not reported	Galeazzi et al., 1999
Freshly prepared Aβ40 (100 μM) Fibrillar Aβ40 (100 μM) obtained after 4.5 days stirring of freshly prepared Aβ at RT	1. Aβ in PBS pH 7.4 mixed with CuSO_4_ (6.25 μg)/H_2_O_2_ (0.28 μl of a 30% stock solution) at 37^°^C for 2 h 2. Aβ in PBS pH 7.4 mixed with CuCl_2_ (38 μg)/ascorbic acid (240 μg) at RT for 2 h 3. Aβ in water irradiated with UV bulb in a glass tube for 24 h 4. Aβ in Na_2_B_4_O_7_ pH 9 mixed with HRP (12.5 μg)/H_2_O_2_ (1.5 μl of a 1% stock solution) at 37^°^C for 2 h	Formation of high molecular weight DiY cross-linked assemblies DiY formed in higher quantity in fibrils than in soluble species CuSO_4_-H_2_O_2_ and HRP- H_2_O_2_ systems more efficient in inducing DiY	Yoburn et al., 2003
Aβ28 (10 μM), Aβ40 (5 μM), and Aβ42 (5 μM)	Aβ in PBS pH 7.4 mixed with CuCl_2_ (25 μM)/H_2_O_2_ (250 μM) for 0–3 days at 37°C	DiY cross-linked Aβ peptides aggregated to dimers and oligomers. The level of DiY cross-linking increased when CuCl_2_ was mixed with H_2_O_2_. DiY cross-linked Aβ were SDS-resistant	Atwood et al., 2004
Aβ42 (10 μM)	Aβ in PBS pH 7.4 mixed with CuCl_2_ (25 μM)/H2O2 (250 μM) with stirring for 22 h at 37°C	Formation of toxic DiY cross-linked dimers and oligomers. Non-toxic dimers and oligomers also formed on Aβ42 with Y to A substitution	Barnham et al., 2004
Aβ28 (3 μM)	Aβ in ammonium acetate buffer pH 7.4 mixed with CuCl (31 μM) or CuCl_2_ (31 μM)/H_2_O_2_ (30 μM) with or without methionine for 24 h at 37°C	Methionine oxidation promotes Y10 oxidation	Ali et al., 2005
Aβ40 (0.4 mg/ml), Aβ28 (0.2 mg/ml)	Aβ in acetate buffer pH 7.4 mixed with Horseradish peroxidase (0.4 mg/ml)/H_2_O_2_ (30 μM) for 6 h at 37°C	Formation of DiY cross-linked dimers and oligomers	Ali et al., 2006
Aβ42 (10 μM)	Aβ in PBS pH 7.4 mixed with CuCl_2_ (10 μM)/H_2_O_2_ (250 μM) with stirring at 200 RPM for 2 or 24 h at 37°C	Aβ42–copper complex formed via a histidine-bridged dimer and DiY cross-linking of Aβ42 show toxicity to cells	Smith et al., 2006
Aβ42 (10 μM)	Aβ in Sodium Phosphate buffer pH 7.4 mixed with CuCl_2_ (10–100 μM)/H_2_O_2_ (250 μM) with stirring at 200 RPM for 24 h at 37°C	High copper-Aβ42 ratio results in a higher level of DiY cross-links and the formation of non-amyloidogenic aggregates. DiY cross-linking forms after the onset of aggregation, not before	Smith et al., 2007b
Aβ42 (5 μM)	Aβ in PBS pH 7.4 mixed with CuCl_2_ (25 μM)/H_2_O_2_ (250 μM) for 5 days	DiY cross-linked Aβ significantly induce cofilin-actin rods in dissociated neuronal culture	Davis et al., 2011
Aβ40 (no concentration given)	Chemical synthesis of DiY-linked Aβ dimers	Cross-linked Aβ showed significantly reduced aggregation kinetics, leading to the formation of long-lived, soluble oligomeric aggregates that reduce cell viability in neuroblastoma cells	Kok et al., 2013
Aβ42 (20 μM)	Aβ in PBS pH 7.4 mixed with CuCl_2_ (20 μM)/H_2_O_2_ (0.5 mM) for 3 days at 37°C with agitation	Cross-linking stabilize Aβ42 assemblies but also appears to slow assembly. Externally administered Aβ42 become oxidized and cross-linked during incubation in neuroblastoma cells, and can be internalized into lysosomes. DiY cross-linked Aβ fibrils are resistant to formic acid digestion	Al-Hilaly et al., 2013
Aβ40 (40 μM)	Aβ40 in ammonium bicarbonate buffer pH 8.5 mixed with horseradish peroxidase (2.2 μM)/H_2_O_2_ (250 μM) overnight at 37°C. Toxicity experiments in SEC isolated Aβ in phosphate buffer pH 8	DiY cross-linked Aβ40 does not aggregate even at 96 h, unless agitated. When agitated, its aggregation rate is ∼15-fold lower than that of non-crosslinked Aβ40. Aggregated DiY cross-linked Aβ40, not monomer/dimer precursors, inhibit LTP similar to aggregated Aβ40. 25 mM ammonium bicarbonate buffer at pH 8.0 and low temperature (4 C) reduces the aggregation of DiY cross-linked and uncross-linked peptides	O’Malley et al., 2014
Aβ40 (30 μM)	Horseradish peroxidase (45 μg/ml)/H_2_O_2_ (20 μM) for 20–30 min	DiY cross-linking stabilize compact dimeric and trimeric Aβ species thus leading to a decrease in the population of more extended species	Sitkiewicz et al., 2014
Aβ42 (40 μM)	Aβ in ammonium bicarbonate buffer, pH 8.5 mixed with Horseradish peroxidase (2.2 μM)/H_2_O_2_ (250 μM) for 14 h at 37°C	DiY cross-linking slows aggregation lag phase and growth phase and forms smaller soluble aggregates than non-oxidized Aβ42	O’Malley et al., 2016
Aβ42 (20 μM), Aβ40 (20 μM)	1. For copper and h202 induced crosslinking of unmodified pro teins (CHICUP), Aβ in phosphate buffer pH 7 mixed with CuCl_2_ (20 μM)/H_2_O_2_ (0.5 mM) for 10 min at 37°C with agitation 2. For PICUP, Aβ in phosphate buffer pH 7 mixed with 1 mM [Ru(bpy)_3_]^2+^ and 20 mM ammonium persulfate (APS) in sodium phosphate buffer pH 7.4, then irradiated using a XGY-II (B) cold halogen light source for 1 s at a distance of 10 cm	DiY cross-linking with lower Cu^2+^/H_2_O_2_ concentrations leads to efficient dimer/oligomer formation and cross-linking with higher Cu^2+^/H_2_O_2_ concentrations result in a decrease in Aβ oligomers Both CHICUP and PICUP DiY cross-linking appear to stabilize Aβ40 and Aβ42 early assemblies (oligomers) and inhibits their conversion into fibrils. The DiY cross-linked peptide disrupts membrane integrity	Williams et al., 2016
Aβ40 (10 μM)	1. Aβ mixed with CuCl_2_ (5 μM)/H_2_O_2_ (50 μM–1.6 mM) in a solution with 100 μM HEPES buffer and 160 mM NaCl for 0–400 h 2. CuCl_2_ (5 μM)/Ascorbate (50–500 μM) in a solution with 100 μM HEPES buffer and 160 mM NaCl for 0–400 h	DiY cross-linking inhibits assembly and causes fiber fragmentation. Inhibition of assembly is more pronounced in a highly oxidative reaction	Gu et al., 2018
Aβ40 (30 and 50 μM)	1. 30 μM Aβ in HEPES pH 7.4 mixed with 30 μM CuCl_2_, 30 μM and 600 μM H_2_O_2_ at 37°C 2. 50 μM Aβ n HEPES pH 7.4 mixed with 50 μM CuCl_2_, and 1 mM H_2_O_2_ at 37°C with agitation a 700 RPM	DiY cross-linking completely inhibited aggregation revealed by Th-T assay	Vázquez et al., 2019
Aβ42, variant Aβ42 (vAβ42) (50 μM)	1. Aβ or vAβ in phosphate buffer pH 7.4 mixed with 400 μM CuCl_2_ or 400 μM CuCl_2_ and 2.5 mM H_2_O_2_ at 37°C 2. Aβ or v Aβ in phosphate buffer pH 7.4 irradiated with ultra-violet-C (UV-C) in the dark with samples kept on ice	DiY cross-linking with CuCl_2_ or UV-C led to Aβ or vAβ stabilization at the time of oxidation. For example, oxidation on oligomers led to oligomer stabilization Seeding experiments revealed that oxidized Aβ are unable to seed further assembly DiY-stabilized Aβ assemblies lost the ability to cause toxicity to neuroblastoma cells	Maina et al., 2020b

Based on these studies (Table 1), it is difficult to compare the levels of DiY produced by the different conditions reported in the literature. This may partly be responsible for the difference in the outcome of DiY cross-linking from the different studies. It is also likely that the dosage of DiY formed per condition would be critical for the assembly of Aβ. This may explain why in some studies, the DiY cross-linked Aβ assembled to fibrils, albeit in a significantly slower fashion (Kok et al., 2013; O’Malley et al., 2014). While in other studies, DiY cross-linking completely inhibited the formation of such higher assemblies (Gu et al., 2018; Maina et al., 2020b).

Moreover, the cross-linking timing may be another factor that would impact the assembly of Aβ. Indeed, it was suggested that DiY cross-linking inhibits primary nucleation and subsequently retards fibril elongation and fibril–fibril interactions (O’Malley et al., 2014). We also showed that DiY cross-linking before the onset of aggregation leads to the rapid self-association of the random-coil rich monomeric assemblies of Aβ into stable amorphous-like structures that never assemble into amyloid fibrils (Maina et al., 2020b). Cross-linking after the onset of aggregation also significantly inhibited Aβ elongations into fibrils and stabilized the intermediate Aβ species (Maina et al., 2020b). Based on our work, it appears like the self-association of Aβ assemblies caused by the DiY cross-linking depletes the pool of available monomers that are needed for the primary and secondary nucleation.

The available data in the literature is yet to clearly define whether DiY cross-linking on Aβ leads to a deleterious effect. This is specifically reflected in the data on toxicity experiments with DiY cross-linked Aβ (Table 1). Some of the studies that suggested that DiY cross-linked Aβ42 dimers and oligomers were toxic did not fully characterize the self-assembly property of the Aβ (Barnham et al., 2004; Davis et al., 2011). Other studies have shown that dimers, trimers, and tetramers stabilized by Aβ oxidation using photo-induced cross-linking of unmodified proteins (PICUP) are toxic to cultured cells (Ono et al., 2009; Jana et al., 2016; Cline et al., 2019). Work from the Walsh group suggested that the toxicity of the DiY cross-linked assemblies was not due to the dimers but the higher assemblies formed by the dimers (O’Malley et al., 2014). Our recent series of work has made a strong case for self-assembly process as a critical driver of Aβ toxicity (Marshall et al., 2016, 2020; Vadukul et al., 2017, 2020; Maina et al., 2020b). The DiY cross-linked dimers reported by O’Malley et al. (2014, 2016) retained their self-assembly properties, albeit at a slow rate. These DiY cross-linked dimers were reported to self-associate to form larger structures and generate aggregates that potently block LTP (O’Malley et al., 2014). In another study, it was shown that toxic DiY cross-linked Aβ assemblies retain their self-assembling property, but at a significantly reduced speed that results in the formation of long-lived, soluble oligomeric aggregates (Kok et al., 2013). This has led to the assumption that DiY cross-linking may stabilize toxic oligomers and prolong their toxicity (Kok et al., 2013; O’Malley et al., 2016; Williams et al., 2016). However, our recent work revealed that DiY cross-linking trapping of Aβ oligomers, a mixture of oligomers and protofibrils and fibrils in a non-assembling conformation renders these assemblies non-toxic to differentiated neuroblastoma cells (Maina et al., 2020b). While non-oxidized Aβ was more toxic during its self-assembling (oligomeric) state compared to when the assembly plateaus (Maina et al., 2020b). We have previously designed a variant form of Aβ that lacks the propensity to aggregate due to F19S and G37D substitutions in Aβ42 (Marshall et al., 2016). Interestingly, the DiY cross-linked non-assembling variant Aβ showed no toxicity to cells (Maina et al., 2020b). Our work suggested that the self-assembly process, not individual Aβ assemblies or DiY cross-linking was responsible for the Aβ toxicity. If DiY Aβ is genuinely toxic, then it may depend on the DiY dose. However, none of the previous studies that evaluated DiY Aβ toxicity except (Maina et al., 2020b) quantified the level of DiY formed on Aβ. As a result, discrepancies may also arise from the differences in the amount of Aβ DiY cross-links used in toxicity assays.

At this stage, it is difficult to conclude the exact role of DiY cross-linking on Aβ aggregation or toxicity in a physiological environment and clearly more studies are needed. This raises the need for maintaining a closely similar method of preparations for Aβ experiments, as the method of peptide preparation, peptide concentration, time and aggregation, and model system used may play a huge role in determining the role of DiY on Aβ toxicity (Cecchi et al., 2008; Krishtal et al., 2015; Jana et al., 2016; Kaniyappan et al., 2017).

On the other hand, DiY cross-linking on existing fibrils might stabilize and help to maintain insoluble Aβ species found in plaques in the AD brain (Wang et al., 1999; Rambaran and Serpell, 2008; Masters and Selkoe, 2012). We have shown previously that DiY antibody colocalizes with Aβ fibrils in human AD plaques (Al-Hilaly et al., 2013). Overall, more work is needed to fully understand the role of DiY on Aβ species *in vitro* and *in vivo*. However, it is also worth noting that many modifications may occur on Aβ e.g., oxidation of histidine, lysine, and methionine35 of Aβ (Palmblad et al., 2002; Kowalik-Jankowska et al., 2004; Ali et al., 2005), which may also affect the Aβ differently (Smith et al., 2007a). Ultimately, studies that define these different modifications and specifically isolate the contribution of DiY would provide the much-needed data to support the interpretation of the studies generated thus far in the literature.

## Tau protein

**FIGURE 3:**
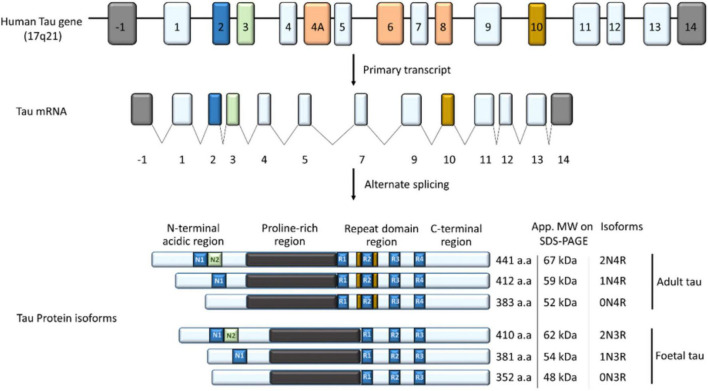
The tau gene has 16 exons; exon 1, 4, 5, 7, 9, 11, 12, and 13 (light blue) are constitutively transcribed in the CNS (Martin et al., 2011). Exon 4A, 6, and 8 (orange) are rarely expressed in the brain but included in mRNA of most peripheral tissues, while exon 14 forms part of the 3′ untranslated region of the tau mRNA (Andreadis, 2005; Connell et al., 2005). Alternate splicing of exon 2 (blue), 3 (Green), and 10 (Yellow) in the CNS generates the widely known six isoforms of tau; 352–441 amino acids in length and 48–67 kDa on SDS-PAGE (Martin et al., 2011). Depending on the inclusion and/or exclusion of exon 2, 3, and 10, tau has zero, one or two (0/1/2) N-terminal inserts and three or four (3R/4R) microtubule binding repeats, leading to the six isoforms of tau in the CNS. Structurally, tau is subdivided into an N-terminal acidic region; proline-rich region/domain (PRD), repeat domain region, and a C-terminal region.

Tau is a small molecular weight protein with the capacity to promote microtubule assembly *in vitro* (Weingarten et al., 1975). It is found in both neuronal and non-neuronal eukaryotic cells, but predominantly in neurons (Loomis et al., 1990; Stoothoff and Johnson, 2005; Rossi et al., 2008; Martin et al., 2011). Tau is well-known for its role in stabilizing the microtubules through their nucleation and elongation (Cleveland et al., 1977). However, recent evidence suggest that tau is a multifunctional protein, playing a role in many cellular compartments, including the synapse and nucleus (Buée et al., 2000; Dixit et al., 2008; Ittner et al., 2010; Martin et al., 2011; Bukar Maina et al., 2016; Maina et al., 2018a,b). Tau is a product of the microtubule-associated protein gene, located on chromosome 17q21.1 (Neve et al., 1986; Andreadis et al., 1992; Andreadis, 2005; Figure 3). A complex post-transcriptional processing of the tau transcript results in a less abundant 2kb tau transcript which encodes for a tau mainly targeted to the nucleus (Wang et al., 1993); 6kb transcript which tau predominantly directed to the soma/axons in the central nervous system (CNS) (Andreadis, 2005; Liu and Götz, 2013); and 8/9 kb transcript producing a tau preferentially expressed in the retina and peripheral nervous system (PNS) and with an apparent molecular weight of about 110–120 kDa, often called high molecular weight tau (Georgieff et al., 1993; Nunez and Fischer, 1997).

The alternate splicing of exon 2, 3, and 10 of the Tau gene generates the six major isoforms of tau in the CNS with three (3R) or four (4R) microtubule-binding repeats on its C-terminal (3R) (Figure 3; Buée et al., 2000; Martin et al., 2011). Structurally, tau is subdivided into four regions; an N-terminal acidic region, a proline-rich domain (PRD), microtubule-binding repeat domain region (MBD), and a C-terminal region, and the epitopes across these areas vary depending on the tau isoform (Buée et al., 2000; Martin et al., 2011; Figure 3).

Physiologically, Tau is a monomeric protein that exists in solution in a random-coil conformation (Schweers et al., 1994). Thus, its aggregation is believed to be a sign of pathology thought to be driven by changes in its conformational state, moving from a random coil to the amyloid cross-β structure (von Bergen et al., 2005). This pathological folding of Tau is evident in a group of diseases collectively called tauopathies, which include AD, Pick’s disease, Huntington’s disease, and Fronto-Temporal Dementia with Parkinsonism linked to Chromosome 17 with MAPT mutations (FTDP-17T) (Oakley et al., 2020). For example, in AD, tau misfolds into paired helical filaments (PHFs) and straight filaments (SFs) and these filaments accumulate in the cell bodies of neurons as neurofibrillary tangles (NFT) (Grundke-Iqbal et al., 1986b; Wischik et al., 1988b). NFT burden correlates with the extent of AD pathology (Arriagada et al., 1992) and provides a reliable staging of the disease process (Braak and Braak, 1991). The precise mechanism involved in tau filament formation is still not fully understood. However, it involves the aggregation of Tau monomers to dimers, oligomers, some of which convert to fibrils, and eventually to PHFs and SFs (Cowan and Mudher, 2013). Many post-translational modifications have been proposed as key molecular events in the abnormal tau aggregation leading to the formation of PHFs. Tau can undergo glycosylation, glycation, prolyl-isomerization, polyamination, nitration, oxidation, ubiquitination, sumoylation phosphorylation (Martin et al., 2011; Guo et al., 2017), and truncation (Wischik et al., 1988b; Wang et al., 2007).

## Dityrosine cross-linking on Tau

Interest in understanding Tau physiology and pathology has resulted in the development of many *in vitro* models over the past decades. These models include the full-length Tau (T40, 2N4R), dGAE (Wischik et al., 1988b), K18, and K19 (Mukrasch et al., 2005; Oakley et al., 2020). The T40 tau which has 441 amino acid residues contains five tyrosine residues, located at positions 18, 29, 197, 310, and 394. However, the model peptides, dGAE, K18, and K19 contain only the tyrosine residue at position 310. Y310 is part of the aggregation prone hexapeptide ^306^VQIVYK^311^ which is thought to be important for Tau assembly *in vitro* (von Bergen et al., 2000; Ganguly et al., 2015). Unlike Aβ, there is a significant lack of research on the role of DiY formation on tau and its contribution to tau pathology. This raises the question as to whether the conclusions drawn from research using Aβ also apply to Tau. Few of the early studies in this area were from the Binder’s laboratory on T40 tau, which represents the full-length tau carrying five tyrosine residues. Specifically, this showed that peroxynitrite oxidation results in the oligomerization of soluble T40 tau via the cross-linking of the tyrosine residues independent of disulfide bonds (Reynolds et al., 2005). However, it was not clear whether DiY formed on tau filaments and the consequence on its aggregation or stability. Tau filaments are usually induced *in vitro* with the help of heparin, arachidonic acid, or other polyanionic molecules (Goedert et al., 1996; Kampers et al., 1996; Pérez et al., 1996; Wilson and Binder, 1997; Friedhoff et al., 1998). Thus, the same group further showed that oxidative stress induced by peroxynitrite on arachidonic acid-induced filaments of T40 tau results in the formation of DiY on the tau filaments. Their work showed that the DiY cross-linking is associated with the stabilization of the pre-formed tau filaments (Reynolds et al., 2006). Although these studies provided the initial evidence that DiY forms in tau, it remained unclear how DiY cross-linking influences tau oligomer formation and aggregation into filaments and NFTs, especially in the absence of additives.

**FIGURE 4:**
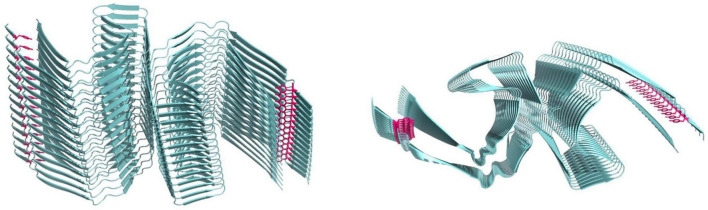
Cryo-electron microscopy structure of paired helical filaments (Fitzpatrick et al., 2017) 5O3L.ENT showing position of tyrosine10 in magenta. Left side view, right panel top view.

We recently established a truncated tau fragment corresponding to residues Ile297–Glu391, called dGAE, as an excellent model of studying tau assembly *in vitro* (Al-Hilaly et al., 2017, 2020). This tau fragment was first isolated from the proteolytically stable core of paired-helical filaments (PHFs) (Wischik et al., 1988b) and more recently was found to overlap with the region identified in the PHF and SF core by cryogenic electron microscopy (CryoEM) (Fitzpatrick et al., 2017; Figure 4). Our work showed that without the addition of exogenous additives dGAE can self-assemble to form filaments *in vitro* that structurally resemble PHF (Al-Hilaly et al., 2017, 2020; Lutter et al., 2022) and more recently, CryoEM structure determination showed the capacity for tau297–391 to form a filament structure identical to those derived from AD brain (Lövestam et al., 2022). Recently, we used MCO and UV oxidation to induce DiY cross-linking in dGAE, showing that oxidation facilitates the assembly of soluble dGAE into T22 antibody-positive tau oligomers which do not elongate into fibrils (Maina et al., 2020a). Different preparations of tau oligomers produced *in vitro* have been described to possess a β-sheet conformation and to induce toxicity in culture (Chen et al., 2009; Lasagna-Reeves et al., 2010; Flach et al., 2012; Tian et al., 2013). Interestingly, we found that the DiY cross-linked tau oligomers formed by dGAE lacked β-sheet structure, instead remained in a random-coil conformation, and were not acutely toxic to differentiated neuroblastoma cells after 3 days of culture (Maina et al., 2020a). This supports the work from the Binder’s group on T40 tau (Reynolds et al., 2005), in showing that DiY cross-linking promotes the formation of tau oligomers. Our work specifically suggests that the oligomers are incapable of further elongation into fibrils and are non-toxic.

Furthermore, we showed that dGAE fibrils formed DiY to a lower extent than the oligomers and other smaller assemblies (Maina et al., 2022a). The reduced ability for these fibrils to form DiY could be due to the reduced accessibility to the tyrosine residue in dGAE (Y310) in the fibrils, unlike in prefibrillar assemblies. Y310 is buried in one of the eight β-sheets of the tau molecule that run along the length of the protofilament, adopting a C-shaped architecture in AD and other tauopathies (Shi et al., 2021; Lövestam et al., 2022; Figure 4). Given the DiY’s ability to enhance protein stability, DiY cross-linked oligomers and fibrils would be expected to have enhanced stability and increased insolubility, similar to tau assemblies extracted from AD brain that show increased insolubility and resistance to proteolytic degradation (Wischik et al., 1988a,b). PHFs are specifically highly insoluble in SDS and sarcosyl (Kondo et al., 1988; Greenberg and Davies, 1990; Lee et al., 1991; Miao et al., 2019). Previous work from the Binder’s group showed that DiY cross-linked full length tau fibrils have increased SDS stability (Reynolds et al., 2006). We also recently showed that DiY cross-linked dGAE oligomers and especially fibrils, display a significantly reduced SDS and heat solubility (Maina et al., 2022a). The fibrils are particularly difficult to resuspend in solution, are heat and SDS-insoluble and transmission electron microscopy show that they maintain strong lateral association (Maina et al., 2022a). These studies suggest that indeed, DiY could promote the stabilization of tau assemblies *in vivo.* This begs the question as to whether DiY forms on tau assemblies *in vivo*? It is known that exposure to reactive oxygen species (ROS), aging, nitrogen dioxide, and lipid hydroperoxides can result in DiY formation (Giulivi and Davies, 1993, 1994; Kato et al., 1994). Oxidative stress is believed to accumulate early on in AD, increasing with the disease pathology (Nunomura et al., 2001; Butterfield and Halliwell, 2019). Thus, such oxidative environment that occurs *in vivo* in AD would favor DiY formation. Indeed, we recently showed that tau oligomers and fibrils extracted from the AD brain are DiY cross-linked (Maina et al., 2022a). However, whether DiY cross-linked tau oligomers also fail to elongate to fibrils *in vivo* is a question of future research. Nonetheless, available data suggests that the DiY cross-linking will promote the characteristic insoluble feature of AD fibrils. There is a significant lack of research on the role of DiY on tau and its implications. More research is needed to fully understand its relevance to AD.

## Could dityrosine cross-linking serve as a biomarker in Alzheimer’s disease?

The chemical stability of DiY could serve as a suitable biomarker for oxidative stress. DiY remains unchanged by exposure to oxygen and low or high pH (DiMarco and Giulivi, 2007). Furthermore, it is highly resistant to acid hydrolysis and proteases (Amado et al., 1984; Giulivi and Davies, 1994). DiY is also not incorporated into *de novo* synthesis of proteins (Eiserich et al., 1999), indicating that the level of DiY will reflect the oxidative damage to endogenous proteins (DiMarco and Giulivi, 2007). In this regard, there has been a strong interest since the 1990s on the possibility of using DiY as a marker of oxidative stress in different diseases. In 1993, DiY was considered an index of organismal oxidative stress (Giulivi and Davies, 1993). In this work, DiY production was shown in red blood cells challenged with the continuous flux of hydrogen peroxide. In the same year, Heinecke et al. (1993a) also showed that DiY could be formed via myeloperoxidase–hydrogen peroxide reaction in human neutrophils and macrophages. For this reason, they suggested that DiY could serve as a suitable marker of radical damage in phagocyte-rich inflammatory lesions *in vivo* (Heinecke et al., 1993b).

In the context of aging, it was found that DiY cross-linked proteins of mouse cardiac and skeletal muscle increased with age (Leeuwenburgh et al., 1997). Leeuwenburgh et al. (1999b) similarly showed that an increase in DiY could be detected through urine assays in aging rats. Such an increase was mirrored by a similar rise in rat skeletal muscle (Leeuwenburgh et al., 1999b). The authors suggested that urine assays of DiY could be used as a non-invasive method of estimating oxidative stress *in vivo* (Leeuwenburgh et al., 1999a,b). Kato et al. (1998) used immunohistochemistry in the human brain tissue to reveal the presence of DiY in lipofuscin pigments from aged human brains. Their work showed a significant elevation of DiY cross-links with age, suggesting a role for it in aging and lipofuscin accumulation (Kato et al., 1998). High levels of DiY was seen in lipofuscin pigments in AD human brains (Al-Hilaly et al., 2019).

In the context of neurodegenerative diseases, increased levels of DiY has been shown in the PD mouse model of 1-methyl-4-phenyl-1,2,3,6-tetrahydropyridine (MPTP) injection (Pennathur et al., 1999). In this model, DiY and nitrotyrosine were specifically increased in the striatum and midbrain, but not in brain regions resistant to MPTP. The authors suggested that oxidative species, including hydroxyl radicals, tyrosyl radicals or peroxynitrite might mediate the damage caused by MPTP to dopaminergic neurons (Pennathur et al., 1999). Similarly, Hensley et al. (1998) measured DiY and nitrotyrosine level in the AD brain regions compared to controls. They revealed that DiY and nitrotyrosine are significantly increased in the hippocampus and neocortical regions of the AD brain and ventricular cerebrospinal fluid compared to levels in controls (Hensley et al., 1998). Their work provided evidence of the suitability of DiY as a biomarker for AD although the sensitivity and reproducibility of the method they utilized—HPLC with electrochemical array detection (HPLC-ECD), has been questioned by others (Duncan, 2003). However, another report using HPLC with fluorescence detection in 63 confirmed AD patient plasma samples revealed that DiY levels were significantly increased compared to controls (Polidori et al., 2004). A similar increase, albeit not significant compared to controls, was observed in the plasma samples from vascular dementia patients (Polidori et al., 2004). DiY cross-linking can result in Aβ dimer formation (Galeazzi et al., 1999; Yoburn et al., 2003; Al-Hilaly et al., 2013). Interestingly, an increase in the plasma level of Aβ dimers was recently shown in AD, serving as a potential biomarker (Villemagne et al., 2010). This further suggests a potential role DiY cross-linking that could help in AD biomarker discovery.

Using immunogold labeling, we have previously shown an increased co-localization of DiY antibody and Aβ antibody in Aβ plaques (Al-Hilaly et al., 2013). Using the same method, we showed an increase in DiY labeling in the CSF samples from AD patients compared to age-matched controls (Al-Hilaly et al., 2013). Although more studies are needed to establish DiY as a biomarker for AD, these studies suggest its utility as a potential biomarker. It would be interesting if future studies investigate whether an increase in DiY shows a disease-dependent rise in the CSF from AD patients. Given the recent promise in plasma-based markers for AD (Karikari et al., 2020a,b; Rodriguez et al., 2020; Suárez-Calvet et al., 2020; Thijssen et al., 2020; O’Connor et al., 2021), it would be useful to expand previous studies on DiY detection in plasma from AD patients (Polidori et al., 2004), to examine whether DiY levels could predict disease progression. This could especially be promising, giving that DiY is a general marker of protein oxidation, which is established to substantially increase in AD (Conrad et al., 2000; Butterfield and Kanski, 2001; Nunomura et al., 2001; Castegna et al., 2003; Polidori et al., 2004; Butterfield and Halliwell, 2019). As such, the DiY could be on multiple proteins, not just Aβ and Tau, which would increase its detection with disease progression.

## Discussion

There are many unanswered questions about the role of DiY on Aβ and Tau which would only become clearer in the future. One of the burning questions is whether DiY cross-linking is good or bad in AD? Given the well-established role of DiY in tissue stability, it may serve to strengthen proteins or tissue following injury in AD. What is DiY’s *in vivo* impact on Aβ and Tau for disease development or progression? Multiple groups have tried to address some of these questions, but the results have been variable, partly due to the differences in the peptide model used, methods of peptide preparation, peptide concentration and aggregation, and experiment duration. Importantly, many oxidative modifications can co-occur on both Aβ and Tau which may also affect them differently. Ultimately, studies that define these different modifications and specifically isolate the contribution of DiY would provide the much-needed data to support the interpretation of the studies generated thus far in the literature. Together, this will be essential for understanding the specific role of DiY on Aβ and tau in AD and its utility for bio-marker or drug discovery.

## Author contributions

All authors listed have made a substantial, direct, and intellectual contribution to the work, and approved it for publication.
